# Genome-wide association study of individual sugar content in fruit of Japanese pear (*Pyrus* spp.)

**DOI:** 10.1186/s12870-021-03130-2

**Published:** 2021-08-16

**Authors:** Sogo Nishio, Takeshi Hayashi, Kenta Shirasawa, Toshihiro Saito, Shingo Terakami, Norio Takada, Yukie Takeuchi, Shigeki Moriya, Akihiko Itai

**Affiliations:** 1 Institute of Fruit Tree and Tea Science, NARO (NIFTS), 2-1 Fujimoto, Tsukuba, Ibaraki 305-8605 Japan; 2 Research Center for Agricultural Information Technology, NARO, 3-1-1 Kannondai, Tsukuba, Ibaraki 305-8666 Japan; 3 Kazusa DNA Research Institute, 2-6-7 Kazusa-Kamatari, Kisarazu, Chiba 292-0818 Japan; 4 Institute of Fruit Tree and Tea Science, NARO, Morioka, Iwate 020-0123 Japan; 5 Graduate School of Life and Environmental Sciences, Kyoto Prefectural University, 74 Kitainayazuma, Seika, Kyoto 619-0244 Japan

**Keywords:** *Pyrus pyrifolia* Nakai, Acid invertase, ERD6-like sugar transporter, Apple, Genome selection

## Abstract

**Background:**

Understanding mechanisms of sugar accumulation and composition is essential to determining fruit quality and maintaining a desirable balance of sugars in plant storage organs. The major sugars in mature Rosaceae fruits are sucrose, fructose, glucose, and sorbitol. Among these, sucrose and fructose have high sweetness, whereas glucose and sorbitol have low sweetness. Japanese pear has extensive variation in individual sugar contents in mature fruit. Increasing total sugar content and that of individual high-sweetness sugars is a major target of breeding programs. The objective of this study was to identify quantitative trait loci (QTLs) associated with fruit traits including individual sugar accumulation, to infer the candidate genes underlying the QTLs, and to assess the potential of genomic selection for breeding pear fruit traits.

**Results:**

We evaluated 10 fruit traits and conducted genome-wide association studies (GWAS) for 106 cultivars and 17 breeding populations (1112 F1 individuals) using 3484 tag single-nucleotide polymorphisms (SNPs). By implementing a mixed linear model and a Bayesian multiple-QTL model in GWAS, 56 SNPs associated with fruit traits were identified. In particular, a SNP located close to acid invertase gene *PPAIV3* on chromosome 7 and a newly identified SNP on chromosome 11 had quite large effects on accumulation of sucrose and glucose, respectively. We used ‘Golden Delicious’ doubled haploid 13 (GDDH13), an apple reference genome, to infer the candidate genes for the identified SNPs. In the region flanking the SNP on chromosome 11, there is a tandem repeat of *early responsive to dehydration* (*ERD6*)-like sugar transporter genes that might play a role in the phenotypes observed.

**Conclusions:**

SNPs associated with individual sugar accumulation were newly identified at several loci, and candidate genes underlying QTLs were inferred using advanced apple genome information. The candidate genes for the QTLs are conserved across Pyrinae genomes, which will be useful for further fruit quality studies in Rosaceae. The accuracies of genomic selection for sucrose, fructose, and glucose with genomic best linear unbiased prediction (GBLUP) were relatively high (0.67–0.75), suggesting that it would be possible to select individuals having high-sweetness fruit with high sucrose and fructose contents and low glucose content.

**Supplementary Information:**

The online version contains supplementary material available at 10.1186/s12870-021-03130-2.

## Background

Pears (*Pyrus* spp.) belong to the subtribe Pyrinae of the Rosaceae and are one of the most important fruit crops in temperate regions. The origin of pear is presumed to be in the mountainous regions of southwestern China and to date back to the Tertiary period (65 to 55 million years ago) [1]. Pears that dispersed toward the west became domesticated as European pear (*P. communis* L.), whereas others that dispersed toward the east gave rise to Asian pear species. Asian pears have fruit with crisp, juicy, and sandy texture that is edible just after harvesting. Records of pear cultivation in China have been found from 2000 to 3300 years ago [1, 2], and major cultivated Asian pears are traditionally classified into three species: *P. ussuriensis* Maxim., *P. bretschneideri* Rehder, and *P. pyrifolia* (Burm. f.) Nakai [3, 4]. Among the three species, *P. pyrifolia* is presumed to have been introduced into Japan prehistorically and became the major species in Japan [5]. Previous reports suggested that there were opportunities for ancient cultivar exchange between Japan and eastern China [6, 7], but the varieties currently cultivated in Japan and China are genetically different from each other, suggesting that they have different breeding histories. In Japan, local cultivar ‘Nijisseiki’ has been one of the leading cultivars, and ‘Nijisseiki’ and its relatives have been repeatedly used as parents in breeding programs, suggesting that recent cultivars have narrow genetic diversity [7].

Some fruit traits that are important for pear breeding programs and have been genetically studied are fruit harvesting day, fruit weight, fruit hardness, acid content, and sweetness [8–11]. Among these, sweetness is the most important factor determining fruit quality [9]. Fruit sweetness is controlled not only by the total sugar content but also by individual sugar composition. While sucrose is the major individual sugar in carbohydrate translocation from source to sink in most crops, the Rosaceae are unique in that sorbitol plays an important role in this process [12]. After the sugar loaded into Rosaceae fruit is converted by several enzymes that play a critical role in sugar metabolism during fruit development [12–17], sucrose, fructose, glucose, and sorbitol accumulate in mature Rosaceae fruit. These sugars have different levels of sweetness per unit mass (g): if sucrose is rated 1, then fructose is 1.50–1.75, glucose is 0.70–0.80, and sorbitol is 0.55–0.70 [18–20]. The mechanisms of sugar accumulation and interconversion are important not only in fruit but also in plant storage organs such as sugar beet taproots, sugarcane stems, and potato tubers [21–23]. Whereas increasing sucrose yield and concentration are important breeding objectives in sugar beet and sugarcane, the content of the reducing sugars fructose and glucose during storage affects the culinary quality of potato products such as chips and French fries.

In a study of various Rosaceae species, pear had a large variation in individual sugar contents in mature fruit [24], whereas cultivar collections of apple and peach had less variation [15, 25–27]. Fructose is dominant in the fruit of most apple cultivars [25, 26], while sucrose is dominant in most peach cultivars [15, 27]. QTLs associated with the conversion of sucrose to hexose in mature fruit were identified on chromosomes 1 and 7 in Japanese pear [9]. Simple sequence repeats (SSRs) corresponding to the regions flanking acid invertase genes *PPAIV3* and *PPAIV1* were detected within the QTL intervals. The enzymes encoded by these genes are located in the vacuole, where they catalyze the conversion of sucrose to hexose. Large-effect QTLs that control the conversion of sucrose to hexose were also identified at the similar position on apple chromosome 1 [28]. Moreover, QTLs for soluble solids concentration (SSC) have been mapped on pear chromosomes 2, 4, 5, 6, and 8 [11, 29], though the effects of these QTLs fluctuated from year to year.

Whole-genome duplications are suggested to have occurred in pear and apple, as their genome sequences have extensive syntenic blocks covering much of the chromosomes (2*n* = 2*x* = 34) [30]. In addition to synteny of their whole genomes, these species also have interesting genes and QTLs in common. The *S* haplotypes that control gametophytic self-incompatibility [31] and genes for susceptibility to *Alternaria alternata* [32, 33] are located at the same positions in both genomes. A member of the 1-aminocyclopropane-1-carboxylate synthase (ACS) gene family is related to several important fruit traits including fruit harvesting day, storage ability, and fruit drop [11, 34–36]. QTLs for harvesting date on chromosome 3 were commonly identified in several studies [11, 37–39]. Because of the high similarity between *Pyrus* and *Malus* genome sequences, pear genetic studies have been conducted using advanced apple genome information [9, 40]. The genome of a doubled haploid ‘Golden Delicious’ (GDDH13) composed of 280 assembled scaffolds and arranged into 17 pseudomolecules is now the most widely used reference genome in apple genetic studies [41]. Although draft genomes of Chinese pear, wild pear in China (*P. betulaefolia*), and European pear are available [42–44], a draft genome of Japanese pear has not yet been available.

Several useful DNA markers have been developed and applied in Japanese pear breeding programs: these include DNA markers to identify self-compatibility [45, 46], a molecular marker associated with disease resistance genes [47, 48], and markers associated with fruit harvesting day [8, 9, 11]. Unlike some traits controlled by a single gene or large-effect QTLs, marker-assisted selection (MAS) for traits controlled by multiple minor genes has not been applied in pear breeding programs. Currently, genomic selection (GS) is gaining attention as an efficient breeding method for such traits in fruit trees. GS utilizes predicted breeding values given by prediction models based on genome-wide single-nucleotide polymorphism (SNP) data to enable selection of superior individuals. The potential of GS for use in Japanese pear breeding was assessed by Minamikawa et al. [10], who used 86 varieties (84 Japanese pear, 2 Chinese pear) and 765 F1 trees from 16 breeding populations (full-sib families) genotyped for SNPs to compare the accuracy of genomic prediction obtained using 12 different methods. The mean prediction accuracy for these models was about 0.6 for fruit quality traits, but those for physiological disorders such as heart rot and watercore were low.

In this study, we updated Minamikawa et al.’s [10] study by further evaluating sugar components, by increasing the numbers of cultivars and individuals, and by applying a more powerful genotyping method. Here, we used double-digest restriction-site associated DNA sequencing (ddRAD-Seq) to genotype 106 cultivars and 17 breeding populations (1112 F1 individuals), representing about 40% more genotypes than in the previous study. This method uses two restriction enzymes, which provides an advantage in precise and repeatable selection and enables paired-end sequencing of identical loci across multiple samples using next-generation sequencing, reducing the cost and time for genotyping [49]. To characterize the sugar metabolism in these materials and to eventually increase the content of specific sugars, here we measured individual sugar contents as well as total sugar content. The objective of this study was to identify the QTLs associated with individual sugar contents and some fruit traits, and to infer the candidate genes for these QTLs. Because many important genes and QTLs have been identified in the apple genome, we used it as a reference genome to identify the chromosome positions of QTLs and surrounding genes. We also examined the potential of GS and considered strategies for introducing GS into pear breeding programs. The genotypes and phenotypes obtained would be a source of information for both practical pear breeding programs and GS.

## Results

### Phenotypic distribution of individual sugars

The average contents of sucrose (SUC), fructose (FRU), glucose (GLC), sorbitol (SOR), and total sugar content (TSC) averaged over 1218 individuals (Table 1) were 43.1, 40.6, 14.9, 32.2, and 130.7 mg/ml, respectively. SUC showed the greatest range of phenotypic variation (1.6–117.0 mg/ml, variance 355.6; Table S1). The distributions of FRU, GLC, SOR, and TSC were much narrower: 9.6–70.7 mg/ml (variance 91.9), 0–35.0 mg/ml (variance 64.3), 11.5–56.3 mg/ml (variance 56.5), and 101.3–173.8 mg/ml (variance 125.2), respectively. Fruit harvest time (HarT) ranged over 93 days (July 23 to October 23, mean August 31; Table S1). The range of fruit weight (FruW) was 92.5–1016.1 g (mean 394.2 g), fruit hardness (FruH) was 2.2–9.5 lb (mean 4.8 lb), SSC was 10.3–18.1% (mean 13.8%), and acidity (Aci, measured by pH), was 3.4–5.6 (mean 4.9).

**Table 1:** Populations and cultivars used in this study and the proportion of genetic clusters at *K* = 4

Population ID	Seed parent	Pollen parent	Number of individuals	“orange” cluster	“light blue” cluster	“dark blue” cluster	“green” cluster
523	Akizuki	373-55	106	0.00	0.00	1.00	0.00
538	Akizuki	Hoshiakari	28	0.00	0.66	0.34	0.00
539	Akizuki	450-63	62	0.00	0.01	0.99	0.00
541	Kosui	Hoshiakari	47	0.00	0.68	0.26	0.05
542	Akiakari	450-7	33	0.00	0.30	0.40	0.29
543	Natsushizuku	Hoshiakari	78	0.00	0.71	0.29	0.00
545	Kosui	Inagi	38	0.04	0.04	0.44	0.49
546	Akizuki	Akiakari	36	0.00	0.19	0.54	0.27
547	Akiakari	Okuroku	70	0.00	0.02	0.03	0.95
574	450-63	Chikusui	51	0.00	0.03	0.97	0.00
578	Hoshiakari	Narumi	100	0.00	0.99	0.01	0.00
581	Kanta	Hoshiakari	62	0.51	0.49	0.00	0.00
588	Hoshiakari	Tsukuba49	121	0.00	0.99	0.01	0.00
589	Kanta	Rinka	49	0.77	0.07	0.04	0.12
590	373-055	Kanta	39	0.56	0.00	0.44	0.00
591	Tsukuba51	Kanta	74	0.99	0.00	0.00	0.00
592	Shurei	Kanta	72	0.61	0.00	0.39	0.00
595	Osa gold	Chikusui	46	0.12	0.02	0.68	0.18
Cultivar collection			106	0.13	0.14	0.37	0.36

The breeding populations  are the product of about five to seven generations of crossing in a pear breeding program at the Institute of Fruit Tree and Tea Science

Population structure was estimated for each individuals in software ADMIXTURE 1.30  K = 4

Phenotypic correlation coefficients and their significances for all trait combinations were calculated (Table 2). SUC had strong negative correlations with FRU, GLC, and SOR (*r* =  − 0.57, − 0.76, and − 0.42, respectively), and GLC had positive correlations with FRU and SOR (*r* = 0.39 and 0.36, respectively). TSC was correlated positively with SUC, FRU, and SOR (*r* = 0.36, 0.22, and 0.29, respectively) but was not significantly correlated with GLC. TSC had a very strong positive correlation with SSC (*r* = 0.92), indicating that the SSC of juice from mature Japanese pear was composed almost entirely of sugars.

**Table 2:** Correlation coefficients calculated for each pair of traits

	SUC	FRU	GLC	SOR	TSC	HarT	FruW	FruH	SSC	Aci
SUC	1									
FRU	-0.57***	1								
GLC	-0.76***	0.39***	1							
SOR	-0.42***	0.07*	0.36***	1						
TSC	0.36***	0.22***	0.01	0.29***	1					
HarT	0.30***	0.09**	-0.18***	-0.24***	0.30***	1				
FruW	-0.02	0.13***	0.07*	-0.01	0.12***	0.53***	1			
FruH	0.12***	-0.23***	-0.26***	0.08*	-0.13***	-0.07*	-0.24***	1		
SSC	0.50***	0.08*	-0.16***	0.18***	0.92***	0.34***	0.09**	-0.048	1	
Aci	-0.00	-0.09**	0.09*	0.15***	0.08*	-0.18***	-0.00	-0.30***	-0.05	1

*P < 0.05, **P < 0.01, ***P < 0.001

SUC = sucrose content, FRU = fructose content, GLC = glucose content, SOR = sorbitol content, TSC = total sugar content, HarT = harvest time, FruW  = fruit weight, FruH = fruit hardness,  SSC = soluble solids concentration (%), Aci = acidity

### ddRAD genotyping

A total of 1.7 billion reads were obtained from the 1218 individuals (average of 1.4 M reads per individual). After trimming low-quality data and adapter sequences, 94.5% of the high-quality reads were successfully mapped onto the apple GDDH13 reference genome. After selecting SNP loci with VCFtools and removing individual SNP markers with < 25% missing data, we obtained 9011 SNPs. Missing data were imputed using Beagle 4.0, and 7463 SNPs with > 0.8 imputation accuracy were selected. To normalize the SNP density, 3484 tag SNPs were selected from the 7463 SNPs using Haploview.

### Linkage disequilibrium and population structure

**Fig. 1:**
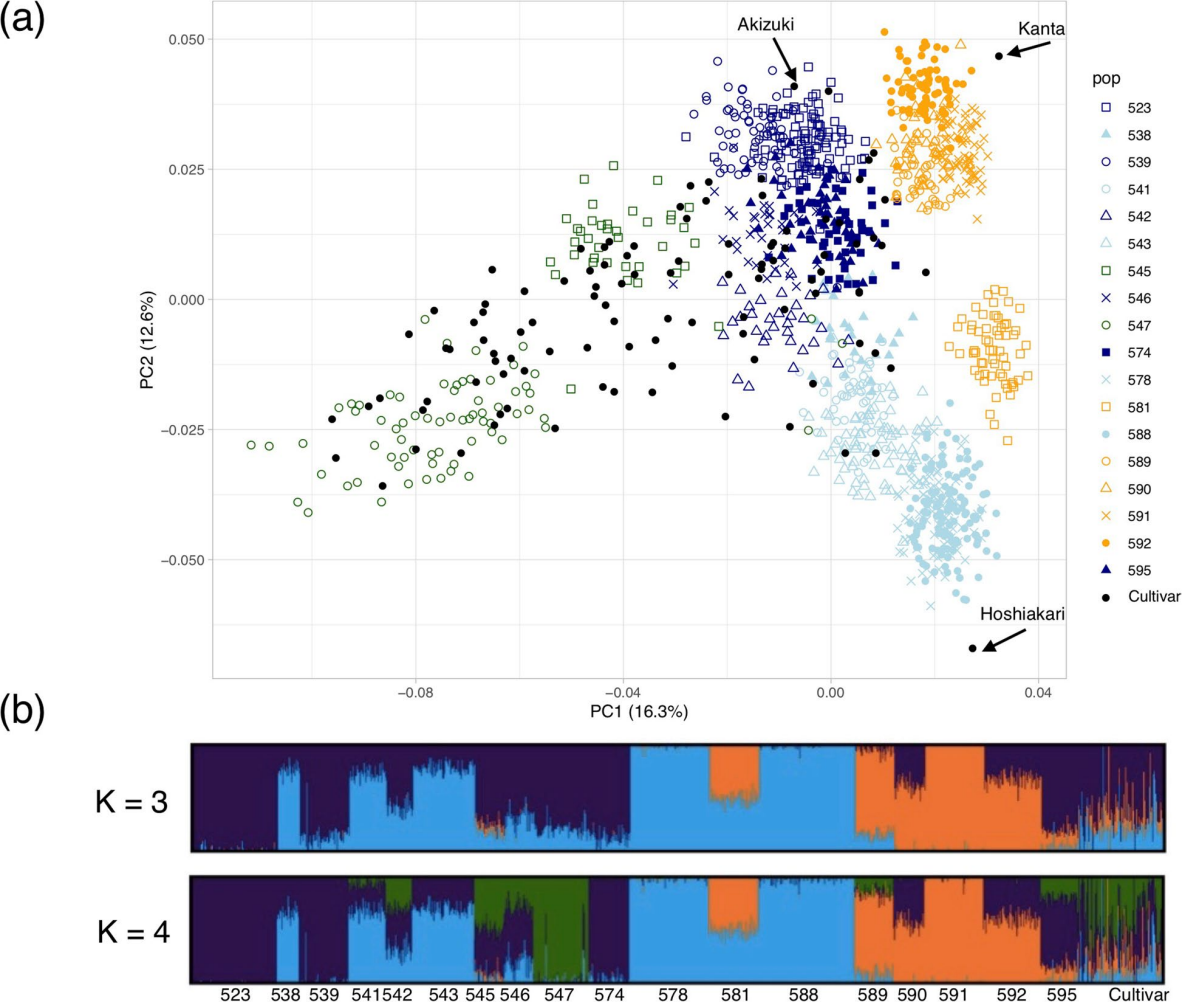
Genetic structure of plant materials used in this study. **a** Principal component analysis was performed in software PLINK v1.90 using 3484 tag single-nucleotide polymorphisms (SNPs). The major parental cultivars of populations in this study (‘Kanta’, ‘Akizuki’, and ‘Hoshiakari’) are indicated by arrows. **b** Population structure estimated for each cross and the cultivar collection in software ADMIXTURE 1.30 at *K* = 3 and *K* = 4. The color used for each population corresponds to the color of its predominant cluster at *K* = 4 in **b**

Linkage disequilibrium (LD) between pairs of loci was estimated using all 7463 SNPs, before selecting the tag SNPs. The *r*^2^ values (estimates of LD) were plotted against marker distances (bp) in the apple genome GDDH13. The average *r*^*2*^ values dropped below 0.2 at a marker distance of 250 kb and below 0.1 at 1750 kb (Figure S1). Population structure was estimated by principal component analysis (PCA) and Bayesian clustering analysis (Fig. 1; Table 1; Table S1). In PCA, the first principal component, which explained 16.3% of the total variation, reflected the difference between old cultivars or populations derived from relatively old cultivars (green) and populations derived from crosses among new cultivars (light blue, orange, and dark blue). The second principal component, which explained 12.5% of the total variation, reflected the genetic difference between ‘Kanta’ and ‘Hoshiakari’, i.e., the populations derived from ‘Kanta’ (orange) were distributed towards positive values of the second principal component and those of ‘Hoshiakari’ (light blue) were distributed towards negative values.

Prior to setting the value of *K* for Bayesian clustering analysis, the standard errors of the cross-validation error were calculated and found to plateau around *K* = 15. Because the cluster diagrams for values of *K* around *K* = 15 were too complicated to provide a clear overview of the genetic relationships among the breeding populations, Bayesian clustering analyses at *K* = 3 and *K* = 4 are shown in Fig. 1. We chose these values because the clusters at *K* = 3 and *K* = 4 roughly corresponded to the classification based on PCA. Although cultivars showed admixed genetic structure, each breeding population had a simple genetic structure. Populations derived from crosses with ‘Kanta’ (proportion of “orange” cluster = 1.00) and other cultivars (population IDs 581, 589, 590, 591, and 592) presented the “orange” genetic cluster, those derived from crosses with ‘Hoshiakari’ (proportion of “light blue” = 1.00) and other cultivars (IDs 538, 541, 543, 578, 581, and 588) presented the “light blue” genetic cluster, and those derived from crosses with ‘Akizuki’ (proportion of “dark blue” = 1.00) and other cultivars (IDs 523, 538, 539, and 546) presented the “dark blue” genetic cluster. When *K* = 4, a “green” cluster was formed from the “dark blue” and the “light blue” clusters in some cultivars and populations (IDs 542, 545, 546, and 547). The average ratio of “orange”:“light blue”:“dark blue” in 1218 individuals was 0.20:0.38:0.42 at *K* = 3, and the average ratio of “orange”:“light blue”:“dark blue”:“green” was 0.19:0.33:0.35:0.13 at *K* = 4. The new “green” cluster seemed to be derived from relatively old cultivars such as ‘Okuroku’ (not included in the 1218 individuals) and ‘Inagi’ (proportion of “green” = 0.60) that are genetically somewhat different from recent cultivars.

### Genome-wide association study (GWAS)

GWAS analysis was conducted for 10 fruit traits by applying two methods: one using a mixed linear model (MLM) to test the significance of association between a single SNP and each trait, and the other using a Bayesian multiple-QTL model. The latter was regarded a variational approximation version of the BayesB method [50] in which all SNPs were simultaneously fitted in the model with a variational approximation [51] (hereafter referred to as vBayesB). In the MLM method, each SNP was tested separately, so some SNPs actually linked to QTLs with minor effects were not identified as significant when there are other QTLs with large effects located near the miner effects and concealed their effects the miner QTLs. To compensate for this drawback of the MLM method, we additionally applied the vBayesB method for GWAS, in which multiple SNPs are simultaneously fitted in a statistical model for explaining phenotypic data. Because the vBayesB method is based on a multiple-SNP model, it allows detection of minor-effect QTLs even when closely linked to those with larger effects. In this method, however, the false positive rate might be slightly increased depending on the non-genetic background effects and the threshold value for significance. For covariates controlling background effects, we included the progeny effect specific to each F1 family in the model while we adopted a threshold value of 0.85 for posterior probability for SNP inclusion in the model. These settings for vBayesB were suitable for controlling the false positive rate. We considered the SNPs that were significantly detected with both methods as reliable QTLs affecting traits related to sugar contents. As shown in Figure S2, a substantial proportion of the SNPs significant with the MLM method were also assigned high posterior probabilities of being included in the model with the vBayesB method.

**Fig. 2:**
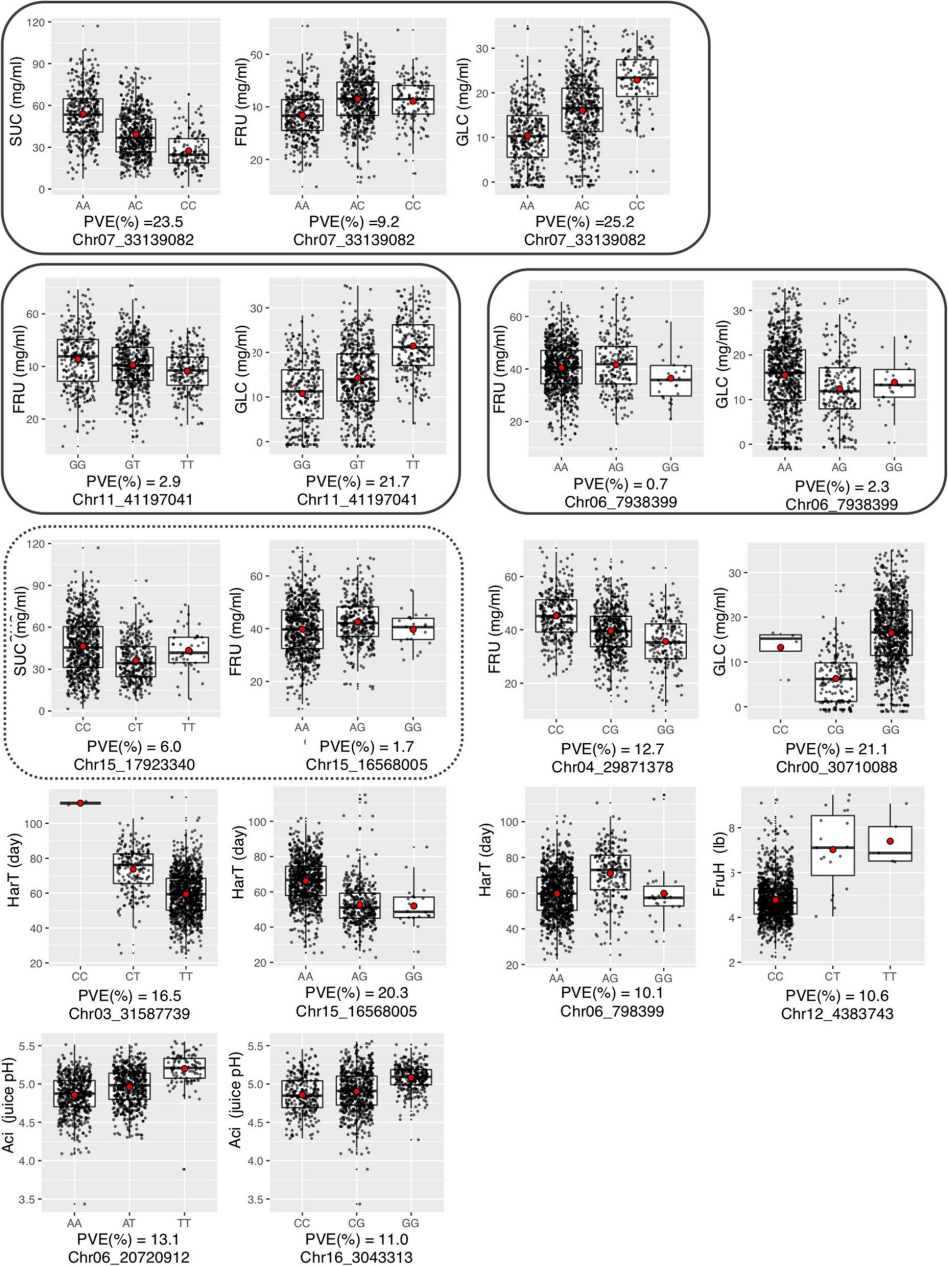
Box plots showing the association of SNP genotypes with six fruit traits. The SNPs shown explained more than 10% of the phenotypic variance for the indicated trait(s). Box plots for different traits associated with the same SNP are enclosed by rounded rectangles. Chr15_1792340 for SUC and Chr15_16568005 for FRU, which are close to one another, are enclosed by a rounded rectangle with a dotted line. Chr04_29871378 for FRU and Chr12_4383743 for FruH were identified only in the vBayesB-based GWAS analysis (Table 4); the rest were identified in the MLM-based GWAS analysis (Table 3) or in both. The fictive chromosome 0 contains all unassigned scaffolds. For each QTL genotype, the red dot and thick horizontal line indicate the average value and median value, respectively

In the MLM-based GWAS, 16 significant SNPs were identified for five fruit traits (SUC, FRU, GLC, HarT, and Aci; Table 3). No significant SNPs were identified for SOR, TSC, FruW, FruH, or SSC. The relationships between genotype and phenotype for SNPs that explained ≥ 10% of the phenotypic variation for a trait are shown in box plots (Fig. 2). Different sugar content traits that were significantly associated with the same SNP are grouped in Fig. 2 in order to compare the relationships between marker genotype and the content of each individual sugar, possibly revealing SNPs involved in sugar conversion. Two QTLs for SUC were detected on chromosomes 7 and 15; four QTLs for FRU were detected on chromosomes 6, 7, 11, and 15; and four QTLs for GLC were detected on chromosomes 0, 6, 7, and 11. Chr07_33139082 was identified as a significant SNP for SUC, FRU, and GLC, with 23.5%, 9.2%, and 25.2% of the phenotypic variance, respectively, explained by the SNP effects. For QTLs that had a large and significant effect on one or more individual sugar contents, the average values of all sugar content traits for each genotype are shown in Table S2. The average values of genotypes at Chr07_33139082 for AA, AC, and CC were 54.0, 39.1, and 27.4 mg/ml for SUC; 36.8, 43.0, and 42.2 mg/ml for FRU; and 10.4, 16.0, and 22.8 mg/ml for GLC, respectively (Fig. 2, Table S2), suggesting that the effect of the QTL on SUC had strong negative correlations with its effects on both FRU and GLC. On the other hand, this SNP seemed to have little effect on TSC (131.5, 130.5, and 129.5 mg/ml for the average values of AA, AC, and CC, respectively; Table S2). For FRU, two SNPs (Chr06_7938399 and Chr11_41197041) were significant. Chr11_41197041 showed effects with negative correlation between FRU and GLC. The effect of Chr11_41197041 on GLC had the highest − log10 (p) value, with 21.7% of the variance explained by the SNP. The average value for GLC of each genotype at Chr11_41197041 was 10.8 mg/ml for GG, 14.3 mg/ml for GT, and 21.4 mg/ml for TT, whereas the average values for FRU were 42.8 mg/ml for GG, 40.5 mg/ml for GT, and 38.1 mg/ml for TT (Fig. 2, Table S2). On the other hand, this SNP seemed to have a relatively small effect on TSC (129.4, 130.9, and 132.1 mg/ml for the average value of GG, GT, and TT, respectively; Table S2). Chr00_30710088, located on fictive chromosome 0 in the apple GDDH13 genome, was also significant for GLC, but was strongly linked to Chr11_41197041 in several populations (for example, *r*^*2*^ = 1.00 between Chr00_30710088 and Chr11_41197041 for population 589, derived from ‘Kanta’ and ‘Rinka’). Thus, the effect of Chr00_30710088 was excluded from further analysis and discussion. Also, Chr15_17923340, which was significant for SUC, and Chr15_16568005, which was significant for FRU, are close to each other, suggesting that these two SNPs are detecting the same QTL. For HarT, two significant SNPs were identified in the MLM-based GWAS. Chr03_31587739 and Chr15_16568005 showed high − log10 (p) values (8.9 and 12.2, respectively) and explained 16.5% and 20.3% of the phenotypic variance, respectively. The difference between the average values of the CT and TT genotypes was 14 days for Chr03_31587739, while the number of CC individuals was too low to include in the comparison. For Chr15_16568005, the average values of the AG and GG genotypes were comparable (52.7 and 52.0, respectively), but that of AA was 66.2. Out of the four significant SNPs associated with Aci, Chr06_20720912 and Chr16_3043313 showed relatively high percentages of variance explained (13.1% and 11.0%, respectively). The average values for Aci at Chr06_20720912 were 4.85 for AA, 4.96 for AT, and 5.20 for TT (Fig. 2), and those at Chr16_3043313 were 4.85 for CC, 4.90 for CG, and 5.08 for GG (Fig. 2).

**Table 3:** Molecular markers showing association with traits evaluated using a mixed linear model (MLM)

SNP	Trait	Chromosome	Position (bp)	-log10 (p) value	PVE (%)
Chr07_33139082	SUC	7	33139082	9.9	23.5
Chr15_17923340	SUC	15	17923340	4.4	6.0
Chr06_7938399	FRU	6	7938399	5.9	0.7
Chr07_33139082	FRU	7	33139082	6.6	9.2
Chr11_41197041	FRU	11	41197041	6.3	2.9
Chr15_16568005	FRU	15	16568005	5.7	1.7
Chr00_30710088	GLC	0	30710088	8.8	21.1
Chr06_7938399	GLC	6	7938399	4.6	2.3
Chr07_33139082	GLC	7	33139082	6.5	25.2
Chr11_41197041	GLC	11	41197041	18.9	21.7
Chr03_31587739	HarT	3	31587739	8.9	16.5
Chr15_16568005	HarT	15	16568005	12.2	20.3
Chr00_23086705	Aci	0	23086705	5.0	4.4
Chr06_20720912	Aci	6	20720912	15.7	13.1
Chr14_14932973	Aci	14	14932973	4.2	3.0
Chr16_3043313	Aci	16	3043313	7.6	11.0

Traits are as defined in Table 2

PVE indicates the percentage of the phenotypic variance explained by the QTL

SUC = sucrose content, FRU = fructose content, GLC = glucose content, HarT = harvest time,, Aci = acidity

**Table 4:** Molecular markers showing association with traits evaluated using a Bayesian multiple-QTL model (vBayesB)

SNP	Trait	LG	Position (cM)	Prob	PVE (%)
Chr07_33139082	SUC	7	33139082	1.00	23.5
Chr09_18310939	SUC	9	18310940	0.93	8.8
Chr12_26930814	SUC	12	26930814	0.99	2.9
Chr04_29871378	FRU	4	29871378	0.97	12.7
Chr06_35256785	FRU	6	35256784	0.87	0.6
Chr10_8714751	FRU	10	8714751	0.97	0.3
Chr10_31918464	FRU	10	31918464	0.87	6.9
Chr11_41197041	FRU	11	41197040	0.99	2.9
Chr14_29691767	FRU	14	29691768	0.99	5.5
Chr01_27419265	GLC	1	27419264	0.91	1.2
Chr07_33139082	GLC	7	33139082	0.89	25.2
Chr10_31918464	GLC	10	31918464	0.87	8.9
Chr11_41197063	GLC	11	41197064	0.96	23.7
Chr14_28907638	SOR	14	28907638	1.00	0.4
Chr15_4672726	SOR	15	4672726	0.88	0.4
Chr02_2240743	TSC	2	2240743	1.00	2.0
Chr05_43292357	TSC	5	43292356	1.00	2.1
Chr07_9849922	TSC	7	9849922	0.98	6.5
Chr04_5362900	HarT	4	5362900	0.93	3.4
Chr05_7790403	HarT	5	7790403	1.00	0.1
Chr05_31445189	HarT	5	31445188	0.98	1.4
Chr06_7938399	HarT	6	7938399	0.97	10.1
Chr07_20343398	HarT	7	20343398	0.99	0.6
Chr13_37081601	HarT	13	37081600	0.97	1.6
Chr17_4322827	HarT	17	4322827	1.00	1.3
Chr01_30025944	FruW	1	30025944	0.89	4.9
Chr07_33920595	FruW	7	33920596	0.95	1.6
Chr09_1463613	FruW	9	1463613	0.89	2.7
Chr16_4765134	FruW	16	4765134	0.88	0.3
Chr02_29263941	FruH	2	29263940	0.91	4.6
Chr08_6783563	FruH	8	6783563	0.88	2.7
Chr12_4383743	FruH	12	4383743	0.87	10.6
Chr02_19653219	SSC	2	19653220	0.91	0.9
Chr04_30194726	SSC	4	30194726	1.00	0.2
Chr17_5809940	SSC	17	5809940	1.00	8.2
Chr01_26225120	Aci	1	26225120	1.00	1.1
Chr04_9535067	Aci	4	9535067	0.92	2.3
Chr06_20720912	Aci	6	20720912	0.91	13.1
Chr06_32310033	Aci	6	32310032	0.95	0.4
Chr09_15996669	Aci	9	15996669	0.90	0.6

Traits are as defined in Table 2

Prob indicates posterior probability

PVE indicates the percentage of the phenotypic variance explained by the QTL

SUC = sucrose content, FRU = fructose content, GLC = glucose content, SOR = sorbitol content, TSC = total sugar content, HarT = harvest time, FruW  = fruit weight, FruH = fruit hardness,  SSC = soluble solids concentration (%), Aci = acidity

In the vBayesB-based GWAS, 40 SNPs showed posterior probabilities exceeding 0.85 for all 10 fruit traits (Table 4, Table S2). Out of those 40 SNPs, 4 were also significant in the MLM-based GWAS: Chr07_33139082 for SUC and GLC, Chr11_41197041 for FRU, and Chr06_20720912 for Aci. Chr11_41197063 (for GLC) is close to Chr11_41197041, suggesting that they are associated with the same gene. Although the posterior probability values were high, the percentages of phenotypic variance explained by the SNPs were not large for most of the detected SNPs. Out of the 40 SNPs, only 7 had percentages of phenotypic variance explained of ≥ 10%, including the four that were also significant in MLM-based GWAS (considering Chr11_41197063 to be the same locus as Chr11_41197041). In addition to those four, Chr04_29871378 explained 12.7% of the phenotypic variance for FRU, and the average value of each genotype was 45.3 mg/ml for CC, 39.6 mg/ml for CG, and 35.6 mg/ml for GG (Fig. 2). These values were positively correlated with those for TSC (134.2 mg/ml for CC, 129.8 mg/ml for CG, and 127.3 mg/ml for GG; Table S2), though this SNP was not detected in the vBayesB-based GWAS for TSC. Chr06_7938399 (for HarT) and Chr12_4383743 (for FruH; see Fig. 2) each accounted for 10% or more of the phenotypic variance for the indicated trait, but these SNP had minor genotype frequency (< 0.05) and did not segregate in most of the populations.

To illustrate the effects of the 56 SNPs from the two GWAS analyses in each population, the percentages of phenotypic variance explained by SNPs in each population were calculated and displayed in a heatmap (Figure S3). While the SNPs identified in MLM-based GWAS tended to show effects across the 1218 individuals, some SNPs identified in vBayesB-based GWAS showed effects specific to a population. For example, Chr04_5362900 (for HarT) and Chr12_4383743 (for FruH) explained high percentages of variance only in the cultivar collection.

Based on the QTLs and genes previously identified in apple, we searched for candidate genes for fruit traits in the GDDH13 genome (Table 5). For the QTLs associated with individual sugar contents, we found an acid invertase gene, a sucrose synthase gene, and *ERD6*-like genes as candidates on apple chromosomes 4, 7, 11, and 15. For the QTLs for fruit harvesting day, a gene for the NAC18.1 transcription factor was found on chromosome 3, and a member of ACS gene family was found on chromosome 15. An aluminum-activated malate transporter-like gene, *ma1*, was also detected on chromosome 16 as a candidate for acidity. When we searched for the locations of these candidate genes in European pears and wild pear in China by using BLAST + , all of the candidate genes were detected in similar regions of both genomes.

**Table 5:** Summary of candidate genes for detected QTL in apple genome GDDH13 and result of the blast to genome of other pear species

					BLAST to genome of European pear (*P. communis* L. ‘Bartlett’)			BLAST to genome of wild pear in China (*P. betulaefolia*)		
SNP	Trait	Candidate gene in GDDH13	ID	Position of candidate gene in GDDH13	Position	Identity (%)	Bit score	Position	Identity (%)	Bit score
Chr07_33139082	SUC,FRU,GLC	Acid invertase, MdoVIN2	MDP0000377084	Chr7:32184887	Chr7:23975117	93.9	1893	Chr7:24394889	94.0	1892
	SUC	Sucrose synthase 4	MD15G1223500	Chr15:18156590	Chr15:14168216	96.3	3561	Chr15:14346485	96.2	3544
	FRU	Sucrose synthase 4	MD15G1223500	Chr15:18156590	Chr15:14346485	96.3	3561	Chr15:14346485	96.2	3544
Chr04_29871378	FRU	ERD6-like gene	MD04G1211000	Chr4:29523287	Chr4:20641984	93.7	1404	Chr4:25696155	95.1	1467
Chr11_41197041	FRU, GLC	A tandem repeat of ERD6-like genes	MD11G1293100	Chr11:41256007	Chr11:28621080	95.4	4401	Chr11:33736771	95.8	4455
		A tandem repeat of ERD6-like genes	MD11G1293200	Chr11:41260335	Chr11:28626231	95.7	5312	Chr11:33741882	95.2	5230
		A tandem repeat of ERD6-like genes	MD11G1293300	Chr11:41264834	Chr11:28631364	94.9	616	Chr11:33746689	94.9	614
		A tandem repeat of ERD6-like genes	MD11G1293400	Chr11:41270166	Chr11:28638653	95.4	5738	Chr11:33757627	95.9	3607
Chr03_31587739	HarT	NAC18.1 transcription factor	MD03G1222600	Chr3:30696191	Chr3:18264874	96.6	1616	Chr3:25257554	96.8	1626
Chr15_16568005	HarT	ACS gene family	MD15G1203500	Chr15:16180415	Chr15:12799631	96.9	1882	Chr15:12917853	95.0	1773
Chr16_3043313	Aci	Aluminum-activated malate transporter-like gene, *ma1*	MDP0000252114	Chr16:3176495	Chr16:2447780	95.9	2486	Chr16:2478566	96.1	2512

Traits are as defined in Table 2

MDP0000377084 and MDP0000252114 are IDs from the Malus × domestica genome v1.0, which is the previous version of GDDH13

The sequences of candidate genes in GDDH13 were compared to the genome sequences of P. communis L. ‘Bartlett’ and P. betuleafolia using a local installation of blastn 2.7.1

SUC = sucrose content, FRU = fructose content, GLC = glucose content, SOR = sorbitol content, HarT = harvest time, Aci = acidity

### Genomic selection

We attempted to predict the breeding values of individuals with genomic best linear unbiased prediction (GBLUP), in which a polygenic effect included in MLM-based GWAS was regarded as the breeding value of an individual, and with the vBayesB-based method, which was also used in GWAS. We used each F1 family as a tested population and the remaining families and cultivars as the training population, where a prediction model was constructed with both observed phenotypes and SNP genotypes of a training population and the breeding values of each tested population were predicted from SNP genotypes with the constructed model. We calculated correlation coefficients between predicted breeding values and observed phenotypes over all individuals in F1 families (Table 6). The prediction accuracies in GBLUP were higher than those in the vBayesB-based method for SUC, FRU, SOR, TSC, HarT, FruW, FruH, and SSC, whereas the prediction accuracies in GBLUP for GLC and Aci were the same as or slightly lower than those in the vBayesB-based method. GLC showed the highest values of prediction accuracy in both GBLUP and the vBayesB-based method (0.75). TSC, FruW, FruH, and SSC showed lower values of prediction accuracy, especially with the vBayesB-based method (0.39–0.46).

**Table 6:** Prediction accuracies of genomic selection for each trait based on genomic best linear unbiased prediction (GBLUP) and vBayesB-based method

Trait	GBLUP	vBayesB
SUC	0.72	0.69
FRU	0.67	0.60
GLC	0.75	0.75
SOR	0.63	0.58
TSC	0.55	0.39
HarT	0.74	0.65
FruW	0.56	0.46
FruH	0.56	0.41
SSC	0.57	0.45
Aci	0.64	0.66

Values indicate Pearson’s correlation coefficient (*r*) between predicted genotypic values and phenotypic values

## Discussion

Several phenotypic correlations among individual sugars were identified in 1218 individuals and cultivars. SUC had strong negative correlations with both FRU and GLC (*r* =  − 0.57 and − 0.76, respectively), and FRU had a positive correlation with GLC (*r* = 0.39). This result was quite similar to those in previous studies of QTLs for individual sugar traits [9] and genetic differences in individual sugars among leading or promising cultivars [52]. In addition, SOR had a negative correlation with SUC in this study (*r* =  − 0.42). These results suggested that sugar conversion from sucrose to hexose or from sorbitol to sucrose had a strong influence on sugar composition in mature fruit. TSC had quite a strong correlation with SSC, suggesting that it would be possible to use SSC for the first screening of individuals: it is much easier to measure SSC (the amount of sugar in fruit) than TSC, which requires high-performance liquid chromatography. In a previous study, the broad-sense heritability of TSC was only 0.33, whereas those of SUC, FRU, GLC, and SOR were 0.64, 0.69, 0.71, and 0.76, respectively [52]. Segregating TSC into individual sugars would be an effective way to identify the genes associated with sugar accumulation and conversion in mature fruit, because the low heritability of TSC would make genetic analyses of that trait difficult. HarT had positive correlations with FruW, TSC, and SSC, as reported in previous studies [10, 53]. This suggests that it might be difficult to develop early-ripening cultivars with large fruit size and high sugar content. On the other hand, FruH and Aci did not show clear correlations with other fruit traits, suggesting that they are independent and controlled by different genes.

In previous studies, LD values of Japanese pear had been calculated based on genetic linkage maps (centimorgan distance) [8, 10]. In this study, we calculated LD based on the apple physical map, enabling us to compare the LD blocks with those in apple. The average *r*^*2*^ values dropped below 0.2 at 250 kb. Our material had a smaller LD block size than the apples studied by Moriya et al. [54] and Kumar et al. [55], but a larger LD block size than in the study of Leforestier et al. [56]. We expected that the LD in Japanese pear would be high because genetic bottlenecks and breeding history would have increased the extent of LD by eliminating recombinant lineages [8]. Nevertheless, the LD block size was smaller than in several apple genetic studies, suggesting that apple experienced a strong bottleneck similar to that presumed to have occurred during pear breeding and domestication. In the present study, the cultivar collection had admixed structure in Bayesian structure analyses and showed broad distribution in PCA compared to individual populations, whereas each population had smaller genetic diversity than the cultivar collection. In the Bayesian structure analysis at *K* = 4, the populations were roughly divided into four groups: those derived from crosses between ‘Kanta’ and other cultivars (“orange”), those derived from crosses between ‘Hoshiakari’ and other cultivars (“light blue”), those derived from crosses between ‘Akizuki’ and other cultivars (“dark blue”), and cultivars and populations relatively distant from those three cultivars (“green”). ‘Kanta’ has high sweetness, with high TSC and FRU [52], ‘Hoshiakari’ has a scab resistance gene inherited from local cultivar ‘Kinchaku’ [57], and ‘Akizuki’ has a good fruit texture with excellent fruit shape. Since each of these cultivars has specific desirable characteristics, they have been used as parents in Japanese pear breeding programs. In essence, the populations used in this study were based on only 13 founder cultivars [57]. Because of the loss of genetic diversity among modern cultivars [36], the genetic diversity of the populations used in this study was not very large. The genetic structure identified in this study reflects the small genetic differences between the parental cultivars used to create the populations, which basically represent the same gene pool, rather than ancestral populations derived from different gene pools.

In this study, we identified several candidate genes based on the genome sequence of GDDH13. All of these candidate genes were successfully mapped to the whole-genome sequences of European pear and wild pear in China, suggesting that those genes are conserved in the Pyrinae (Table 5). We identified a QTL associated with conversion of SUC to FRU and GLC on chromosome 7. A previous study also identified a QTL associated with conversion of SUC to FRU and GLC in this region (chromosome 7) and on chromosome 1, and further suggested that acid invertase genes *PPAIV1* and *PPAIV3* were the candidate genes [9]. In apple, a similar QTL for conversion of individual sugars was also identified on chromosome 1 in both mature fruit and fruit after storage [28]. Thus, it is possible that the AIV gene family plays an important role in determining individual sugar contents in mature fruit of Rosaceae fruit species. A candidate gene for QTLs that were significant for SUC and FRU on chromosome 15 (Chr15_17923340 and Chr15_16568005) might be sucrose synthase 4 (MD15G1223500, Chr15:18,156,590–18,162,358). Another possibility is sucrose phosphate synthase 1F (MD15G1164900, Chr15:12,407,116–12,413,753), though it is located at a distance of about 500 kb from the detected SNP. Because sucrose synthase catalyzes the reversible conversion of sucrose and UDP to UDP-glucose, and sucrose phosphate synthase catalyzes the synthesis of sucrose from sucrose 6-phosphate, it is reasonable that these genes are related to the QTLs on chromosome 15. Chr11_41197041 was identified as a significant SNP for both FRU and GLC, showing a strong effect in several populations (Table S2). Because a negative correlation between FRU and GLC was observed for this SNP, it would potentially enable us to select genotypes with high FRU and low GLC, thus improving fruit sweetness. The QTL identified at Chr04_29871378 for FRU explained 12.7% of the variance among 1218 individuals and showed relatively high percentages of variance in several populations (more than 10% of the variance explained in nine populations; Figure S3). The effect of the QTL was limited to FRU; other individual sugars seemed to be unaffected, suggesting that an increase in FRU is directly associated with an increase in TSC (Table S2).

Interestingly, *early responsive to dehydration* (*ERD6*)-like gene, encoding sugar transporters, are also located near two of the SNPs (Chr11_41197041 and Chr04_29871378). A single *ERD6*-like gene was detected near Chr04_29871378 (MD04G1211000; Chr4:29,523,287–29,525,923), whereas a tandem repeat of *ERD6*-like genes (MD11G1293100, MD11G1293200, MD11G1293300, MD11G1293400; Chr11:41,256,007–41,275,197) was detected near Chr11_41197041. Arabidopsis ERD6 was found to be sugar transporters in bacteria, yeasts, other plants, and mammals [58]. This transporter was reported to be localized to the tonoplast, acting as an H + /glucose symporter to facilitate the export of glucose from the vacuole to the cytosol and regulating cellular glucose in response to various stresses [59]. Tandem duplications of the *ERD6*-like family genes were found to be conserved in citrus, grape, apple, and Chinese jujube [60]. The order of the tandem repeat of *ERD6*-like genes detected in this study is also conserved in the genomes of European pear and wild pear in China (Table 5). Zhang et al. [61] suggested that duplication of sugar transporter genes plays crucial roles in sugar accumulation. Because Chr11_41197041 was the SNP with the largest effect on GLC, explaining 21.7% of the phenotypic variance, it is reasonable that transport of glucose from the vacuole to the cytosol by this transporter contributed to the observed changes in the individual sugar contents in mature fruit. The vacuole of mature fruit can occupy more than 90% of the cell volume [62], storing sugars and other compounds. Export of glucose instead of H^+^ by the product of this gene may produce an electrochemical gradient across the vacuolar membrane and bring other sugars into the vacuole.

Several QTLs for HarT and Aci similar to those identified here were identified in previous studies of apple. The NAC18.1 transcription factor (MD03G1222600; Chr3:30,696,191–30,698,216) is a promising functional candidate for fruit ripening [63] and is close to Chr03_31587739 (for HarT) in this study. A NAC-family genes were also associated with maturity date and slow ripening in peach [64, 65]. One member of the ACS gene family (MD15G1203500) is located at Chr15:16,180,415, which is close to Chr15_16568005 (for HarT) in this study. This QTL and the function of the ACS gene have already been analyzed in several studies and found to affect fruit harvesting day, storage ability, and fruit drop [11, 34–36]. The early-harvesting genotype is correlated with increased fruit drop and short storage ability, suggesting that pear breeders need to consider carefully whether they use this marker for MAS, depending on their breeding objectives [38]. In apple, an aluminum-activated malate transporter-like gene (*ma1*, MDP0000252114) was identified as the candidate for a gene associated with low fruit acidity [66]. In the apple GDDH13 genome, *ma1* is located at Chr16:3,176,495–3,179,279, which is close to Chr16_3043313 (for Aci) in this study. In apple, two major loci, *Ma3* on chromosome 8 and *Ma1* on chromosome 16, play an important role in fruit acidity [67]. On the other hand, another major locus for Aci in this study was identified on chromosome 6: this locus showed quite a large effect and explained more than 30% of the phenotypic variance in seven populations (Chr06_20720912 in Figure S3).

In this study, we focused on detecting QTLs and inferring putative candidate genes for those QTLs, whereas Minamikawa et al. [10] focused on the accuracy of various methods for genomic prediction rather than on inferring candidate genes. We estimated prediction accuracy for GS using GBLUP and a vBayesB-based method for GWAS analyses. Most traits showed higher prediction accuracies with GBLUP than with the vBayesB-based method; on the other hand, the prediction accuracy for Aci was slightly higher when the vBayesB-based method was used. It is natural that the best GS model varies depending on the trait because the distribution of the trait values vary depending on heritability, allele frequencies, and the population structure of the materials. It will be important to choose the appropriate model for each trait. The prediction accuracies for SUC, FRU, and GLU with GBLUP were relatively high (0.67–0.75), suggesting that it would be possible to select individuals that have high-sweetness fruit (high SUC and FRU, and low GLU). On the other hand, the prediction accuracy for TSC, which is the most important trait determining fruit quality, is not high enough for use in GS (0.55 in GBLUP) There is room to improve the prediction accuracy by constructing the genome sequence of Japanese pear and using it as a reference genome, thus increasing the number of SNPs and improving map precision. The accuracy of genomic prediction can also be improved further when full-sib data for the target family are available [10]. It would be a good idea to apply GS to the populations used in this study, i.e., those derived from the crosses between ‘Hoshiakari’, ‘Kanta’, ‘Akizuki’, and other cultivars. The practical way to introduce genomic selection in breeding programs is to predict the genetic values of each trait of seedings using next-generation sequencing and the best fitting model, and to undercut the seedlings that have lower predicted values before planting in breeding fields. The selecting intensity can be flexibly determined by breeders based on the objective of breeding programs, the number of the seedlings and size of the breeding fields.

## Conclusions

In this study, we collected phenotypic data for fruit traits and conducted GWAS and GS in Japanese pear. Several important QTLs for fruit traits were identified, and genes associated with sugar accumulation were predicted by GWAS using a large number of individuals. The SNP located closest to *PPAIV3* on chromosome 7 and a newly identified SNP at chromosome 11 had large effects on individual sugar contents. The SNP on chromosome 4 that was associated with FRU would be useful for increasing the contents of FRU and TSC without decreasing the contents of other individual sugars. The candidate genes of QTLs identified in this study are conserved in the genomes of several Pyrinae species. Further studies including expression analysis of those genes and developing gene-specific markers would contribute to clarifying the mechanisms of sugar accumulation and validating the candidate genes for fruit traits. Fruit traits are complex and controlled by multiple factors, so it is important to accumulate relevant genetic information. The traits evaluated in this study covered the principal fruit traits in pear breeding programs, so the results obtained illustrate the feasibility of GS for fruit traits in pear.

## Methods

### Plant materials

A cultivar collection including 106 cultivars and 17 breeding populations (consisting of 1112 F1 individuals) were used in this study (Table 1, Table S1). The cultivars were preserved at the NARO (National Agriculture and Food Organization) Genebank (www.gene.affrc.go.jp) and the breeding populations were developed at Institute of Fruit Tree and Tea Science, NARO. The population ID numbers are indicated in Table 1, and each family contained 28 to 121 individuals. Among those materials, 74 cultivars and 498 individuals were the same as those used in Minamikawa et al. [10]. The breeding populations originated from local cultivar ‘Nijisseiki’ and are the product of about five to seven generations of crossing in the NIFTS pear breeding program. The cultivars and F1 individuals were grown with cultural techniques used in commercial production in Japan [68]. The trees were trained on horizontal trellises, pruned annually in winter, and treated for pests and diseases. Fruits were thinned to one fruit per three fruit clusters in mid-May and harvested during late July to early November according to a color chart that indicates the optimum color for picking Japanese pear [69].

Ten fruit traits were evaluated: SUC, FRU, GLC, SOR, TSC, HarT, FruW, FruH, SSC, and Aci. The contents of each sugar (SUC, FRU, GLC, SOR) were analyzed in fruit from each individual. To do this, the juice from two fruits per sampling date was extracted and the samples were combined. Sampling was performed on two days in each of the years that the individual or cultivar was analyzed. The analysis of sugar components was based on the method described by Nishio et al. [9]. TSC was calculated by summing the contents of the four sugars. The harvest date for each fruit was expressed as the number of days after June 30 (i.e., July 1 = day 1), and the average value of harvest date for each fruit was used as its phenotypic score. Fruit weight (g) per fruit (FruW) was measured on a digital scale on each harvest date. Fruit hardness (FruH) was measured by the Magness–Taylor pressure test (lb). Total SSC was determined with a digital refractometer (DBX-55; Atago, Tokyo, Japan) by adding a few drops of juice onto the lens of the measuring device, and the results were recorded as SSC (%). Juice pH was determined with a pH meter (IQ240; Scientific Instruments, San Diego, CA, USA) to estimate acidity (Aci).

Phenotypic data were collected from 2014 to 2018. The majority of individuals and cultivars were evaluated for more than one year. The average phenotypic values over five years (2014–2018) were generated by the allEffects function from the package “effects”. Phenotypic correlation coefficients and their significance were calculated for all trait combinations using R (4.0.0) (R Development Core Team).

### SNP genotyping

Genomic DNA was extracted from young leaves with a DNeasy Plant Mini Kit (Qiagen, Hilden, Germany) according to the manufacturer’s instructions. ddRAD-Seq libraries were constructed as described in Shirasawa et al. [49]. A total of 200 ng of genomic DNA for each individual was double digested with PstI and MspI (FastDigest restriction enzymes; Thermo Fisher Scientific, Waltham, MA, USA), ligated to adapters using the LigaFast Rapid DNA Ligation System (Promega, Madison, WI, USA), and purified using Agencourt AMPure XP (Beckman Coulter, Brea, CA, USA) to eliminate short (< 300 bp) DNA fragments. Purified DNA was diluted with H_2_O and amplified by 20 cycles of PCR with indexed primers. Amplicons were pooled and separated on a BluePippin 1.5% agarose cassette (Sage Science, Beverly, MA, USA), and fragments of 300–900 bp were purified using a Mini Elute Kit (Qiagen). The library was then sequenced using a HiSeq 4000 (Illumina, Inc., San Diego, CA, USA).

SNPs and indels were identified according to Acquadro et al. [70]. Illumina reads were de-multiplexed on the basis of the Illumina TruSeq index. Raw reads were analyzed with Scythe (https://github.com/vsbuffalo/scythe) for filtering out contaminant substrings and Sickle (https://github.com/najoshi/sickle), which removes reads with poor-quality ends (Q < 30). Alignment to the reference apple genome (GDDH13 v1.1; https://www.rosaceae.org/species/malus/malus_x_domestica/genome_GDDH13_v1.1) was carried out using BWA aligner [71] (e.g., mem command) with default parameters and avoiding multiple-mapping reads. SNP and indel mining was conducted by adopting a Samtools-based pipeline [72]. SNPs and indels (hereafter called SNPs) detected from the alignments were filtered with VCFtools (version 0.1.13; parameters: –minQ 20 –minDP 10 –maf 0.01 –maf 0.99 –min-allele 2 –max-allele 2). Individual SNP markers with > 25% missing data were removed from further analysis, and the remaining missing values were phased and imputed using Beagle v4.1 [73]. Only the SNPs with an imputation accuracy > 0.8 were adopted. The tagger software implemented in Haploview (http://www.broad.mit.edu/mpg/haploview) was used to select tag SNPs using its pairwise function, with a minimum *r*^*2*^ of 0.8.

The draft genomes of Chinese pear (*P. bretschneideri*)*,* wild pear in China (*P. betulaefolia*), and European pear *(P. communis)* are all currently available [42–44]. However, the draft genome of Chinese pear was constructed in 2013 and we found that it contains many assembly errors. On the other hand, the genome sequences of European pear and wild pear in China are presumed to have a high level of accuracy, but they are completely different species from *Pyrus pyrifolia.* There is also less information on genes and transcripts and fewer relevant genetic studies in comparison with those using apple reference genomes. Because the apple genome GDDH13 is the most frequently used reference genome in the Rosaceae and it has precise order and rich information on transcripts, QTLs, and LDs, we used GDDH13 as the reference genome in this study.

### LD and population structure

LD was estimated using data after the imputation. Based on the apple GDDH13 genome, the detected SNPs were aligned according to their positions. LD between pairs of SNPs was calculated using R (4.0.0) (R Development Core Team). The SNPs mapped on the fictive chromosome 0, which contains all unassigned scaffolds, were removed to calculate the LDs. Average LD values (*r*^*2*^) were plotted against physical distances in increments of 10 kb.

Population structure was estimated for each cross and for the cultivar collection in ADMIXTURE 1.30 [74] using the 3484 tag SNPs. The software PLINK v1.90 [75] was used to generate an input file from a vcf file. The analysis was run five times for each value of *K* (number of inferred ancestral populations) from 1 to 20 to estimate the cross-validation values. The cross-validation value gradually decreased as the value of *K* increased. The CLUMPAK online tool [76] was applied to graphically display the results produced by ADMIXTURE at *K* = 3 and *K* = 4. PCA was performed in PLINK v1.90. Prior to PCA, an input file was created by conducting linkage pruning using the –indep-pairwise option in PLINK (plink –file data –indep-pairwise 50 10 0.1). PCA was performed using the –pca option in PLINK. The plot was drawn with the R package “ggplot” [77].

### Statistical methods in GWAS

For the MLM-based GWAS, the effect of a SNP and a polygenic effect affected by the genetic background of an individual were included in the model as a fixed effect and a random effect, respectively. The covariance matrix of polygenic effects between individuals was established with a kinship matrix calculated from SNP genotypes. The effects of population structure were also included as fixed effects using the first three principal components obtained from the SNP genotype data. We used the R package rrBLUP ver. 4.3 [78] for this MLM-based GWAS and evaluated the effects of the significant SNPs on the basis of the mean phenotypic value for each genotype of the SNP.

For the vBayesB-based GWAS, a Bayesian multiple-QTL model was applied in which all SNPs were simultaneously fitted in the model assuming a specific probability for each SNP included in the model, called SNP weight, and a specific variance for each SNP effect as well as the progeny effect specific to each F1 family. The progeny effect specific to each F1 family was also included in the model to control for the difference in genetic background between F1 families. The model was fitted with a variational approximation method proposed by Hayashi and Iwata [51] using a custom program written in Fortran. This Bayesian procedure was regarded as a variational approximation of BayesB [51] and was previously applied to GWAS in chestnut [79], in which the adopted estimation methods were described (we refer the reader to this paper for computational details). The settings of hyperparameter values of the prior distributions were conducted following this paper [79].

To validate the effect of the detected SNPs, the average values of each genotype for the SNPs and the distribution of the phenotypic values in the SNP genotypes were plotted. These plots were drawn with the R package “ggplot” [77]. The phenotypic variance explained by each SNP was calculated according to Nishio et al. [36]. The percentage of the phenotypic variance explained by each SNP was calculated by dividing the variance of the SNP by the total phenotypic variance.

To infer the candidate genes of the detected QTLs, we thoroughly checked the QTLs and genes identified in previous apple genetic studies [28, 38–40, 42, 55–57, 63, 66, 67]. If genes related to sugar synthesis, sugar conversion, or sugar transport were located within 200 kb of a detected QTL, they were listed in Table 5. Similarly, if previously identified genes for other fruit traits were located within 200 kb of a detected QTL, they were added to Table 5. BLAST + [80] was used to check whether these genes were located at similar positions in the whole-genome sequences of European pear [42] and wild pear in China [44].

### Accuracies of predicted genomic breeding values in GS

The two methods used in GWAS, MLM-based and vBayesB-based, were also used for prediction of breeding values based on SNP genotypes. In the MLM-based method, a polygenic effect was regarded as a breeding value that was predicted from SNP genotypes through a kinship matrix. This MLM-based method was referred to as GBLUP when applied to GS. In this study, an intercept but no fixed effects were included in MLM for GS. In the vBayesB method, we obtained estimates of a SNP effect and a SNP weight for each SNP, regarded as an approximate posterior probability of each SNP included in the model; accordingly, the predicted breeding value of an individual was calculated as the sum of the estimated SNP effects multiplied by the SNP weights over all SNPs.

Generally, in practical breeding programs, new elite cultivars and selections have been used as parents to create breeding populations and to select genotypes superior to those of the established leading cultivars. Thus, GS would be applied in breeding populations obtained by crossing between new combinations of parental genotypes. To evaluate the accuracy of genomic prediction for practical pear breeding programs, we conducted a cross-validation in the following way. Each F1 family was regarded as a tested family, and the set of cultivars and F1 families with the tested family excluded was regarded as a training population. A prediction model was constructed using data for both phenotypes and SNP genotypes of a training population, and breeding values of individuals of a tested F1 family were predicted with a model using only their SNP genotypes. This process was repeated until all F1 families were selected just once as a tested population and the prediction accuracy was evaluated with the Pearson’s correlation coefficient (*r*) between observed phenotypic values and predicted genotypic values. When the estimated *r* was less than 0, it was regarded as 0. The prediction accuracy for each tested family based on the training population (which excluded the target family) was calculated and averaged to understand the accuracies of predicted genomic breeding values for the breeding populations used in this study.

### Availability of data and materials

The datasets supporting the conclusions of this article are included within the article and its supplementary information files. Sequence reads are available from the Sequence Read Archive (DRA) of DNA Data Bank of Japan (DDBJ) under the accession number of DRA011324 (https://ddbj.nig.ac.jp/DRASearch/submission?acc=DRA011324).

## Supplementary Information

**Additional file 1:Figure S1** Plots of average linkage disequilibrium values (r2) against physical distances in increments of 10 kb. Gray curves show local polynomial fits obtained using kernel smoothing regression. Dotted lines indicate the genetic distance at which r2 fell below 0.2. **Figure S2** Summary of the results of genome-wide association studies using two different models. a Manhattan plots of −log10(p) values based on a MLM for 10 fruit traits. Red lines indicate a false discovery rate of 0.05. Chromosome location information is based on GDDH13 Version 1.1. The fictive chromosome 0 contains all unassigned scaffolds. b Posterior probability of having a QTL based on vBayesB, estimated for 10 fruit traits. **Figure S3** Heatmap showing the percentage of the phenotypic variance explained by each QTL in each population. The percentages for the cultivar collection (designated as “Cultivar”) and for all 1218 accessions including the populations and cultivars (designated as “ALL”) are shown on the right. The prefix rrBLUP indicates the SNPs identified in the MLM-based GWAS analysis; BayesB indicates those identified in the vBayesB-based GWAS. A blank indicates that the population showed no segregation for the SNPs. The intensity of the red color corresponds to the percentage of phenotypic variance explained

**Additional file 2:Table S1** The cultivars and individuals used in this study and the performance for fruit traits.

**Additional file 3:** **Table S2.** Average values of individual and total sugar contents for each genotype of three SNPs having a large and significant effect on at least one trait.

## Abbreviations

Aci: Acidity
ACS: 1-Aminocyclopropane-1-carboxylate synthase
FRU: Fructose content
FruH: Fruit hardness
FruW: Fruit weight
GBLUP: Genomic best linear unbiased prediction
GDDH: ‘Golden Delicious’ doubled haploid
GLC: Glucose content
GS: Genomic selection
GWAS: Genome-wide association study
HarT: Fruit harvest time
MLM: Mixed linear model
NIFTS: Institute of Fruit Tree and Tea Science, NARO
PCA: Principal component analysis
SNP: Single-nucleotide polymorphism
SOR: Sorbitol content
SSC: Soluble solids concentration
SSR: Simple sequence repeat
SUC: Sucrose content
TSC: Total sugar content
