# Supplementary material for: Genome-wide association study of individual sugar content in fruit of Japanese pear (Pyrus spp.)
Source: BMC Plant Biol. 2021 Aug 16;21:378. doi: 10.1186/s12870-021-03130-2 (PMC8369641; doi:10.1186/s12870-021-03130-2)
Supplement: Supplementary file 1 — Additional file 1:Figure S1 Plots of average linkage disequilibrium values (r2) against physical distances in increments of 10 kb. Gray curves show local polynomial fits obtained using kernel smoothing regression. Dotted lines indicate the genetic distance at which r2 fell below 0.2. Figure S2 Summary of the results of genome-wide association studies using two different models. a Manhattan plots of −log10(p) values based on a MLM for 10 fruit traits. Red lines indicate a false discovery rate of 0.05. Chromosome location information is based on GDDH13 Version 1.1. The fictive chromosome 0 contains all unassigned scaffolds. b Posterior probability of having a QTL based on vBayesB, estimated for 10 fruit traits. Figure S3 Heatmap showing the percentage of the phenotypic variance explained by each QTL in each population. The percentages for the cultivar collection (designated as “Cultivar”) and for all 1218 accessions including the populations and cultivars (designated as “ALL”) are shown on the right. The prefix rrBLUP indicates the SNPs identified in the MLM-based GWAS analysis; BayesB indicates those identified in the vBayesB-based GWAS. A blank indicates that the population showed no segregation for the SNPs. The intensity of the red color corresponds to the percentage of phenotypic variance explained [file 12870_2021_3130_MOESM1_ESM.pptx]

## Slide 1
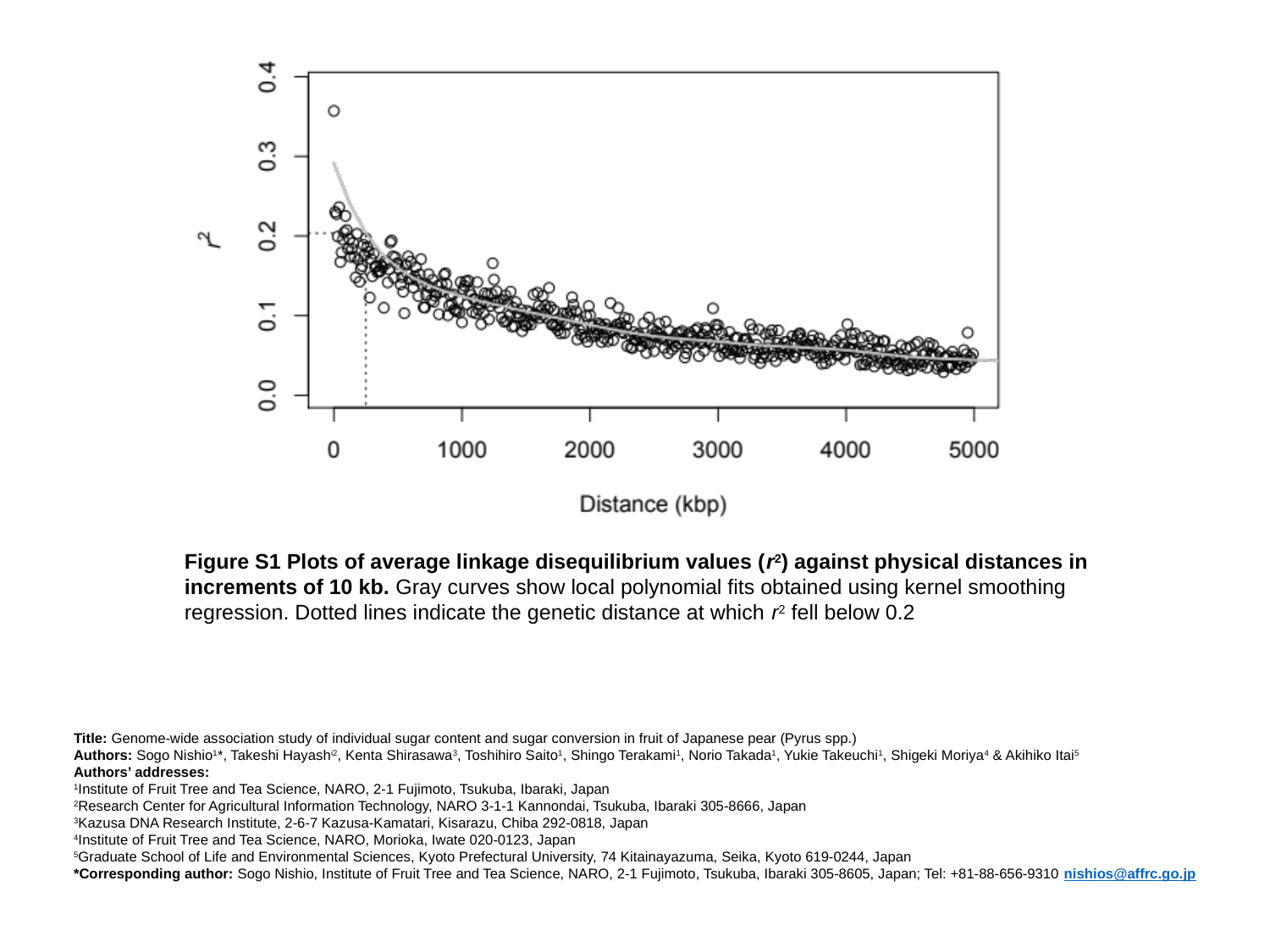

Figure S1 Plots of average linkage disequilibrium values (r2) against physical distances in increments of 10 kb. Gray curves show local polynomial fits obtained using kernel smoothing regression. Dotted lines indicate the genetic distance at which r2 fell below 0.2
Title: Genome-wide association study of individual sugar content and sugar conversion in fruit of Japanese pear (Pyrus spp.)
Authors: Sogo Nishio1*, Takeshi Hayashi2, Kenta Shirasawa3, Toshihiro Saito1, Shingo Terakami1, Norio Takada1, Yukie Takeuchi1, Shigeki Moriya4 & Akihiko Itai5
Authors’ addresses:
1Institute of Fruit Tree and Tea Science, NARO, 2-1 Fujimoto, Tsukuba, Ibaraki, Japan
2Research Center for Agricultural Information Technology, NARO 3-1-1 Kannondai, Tsukuba, Ibaraki 305-8666, Japan
3Kazusa DNA Research Institute, 2-6-7 Kazusa-Kamatari, Kisarazu, Chiba 292-0818, Japan
4Institute of Fruit Tree and Tea Science, NARO, Morioka, Iwate 020-0123, Japan
5Graduate School of Life and Environmental Sciences, Kyoto Prefectural University, 74 Kitainayazuma, Seika, Kyoto 619-0244, Japan
*Corresponding author: Sogo Nishio, Institute of Fruit Tree and Tea Science, NARO, 2-1 Fujimoto, Tsukuba, Ibaraki 305-8605, Japan; Tel: +81-88-656-9310 nishios@affrc.go.jp

## Slide 2
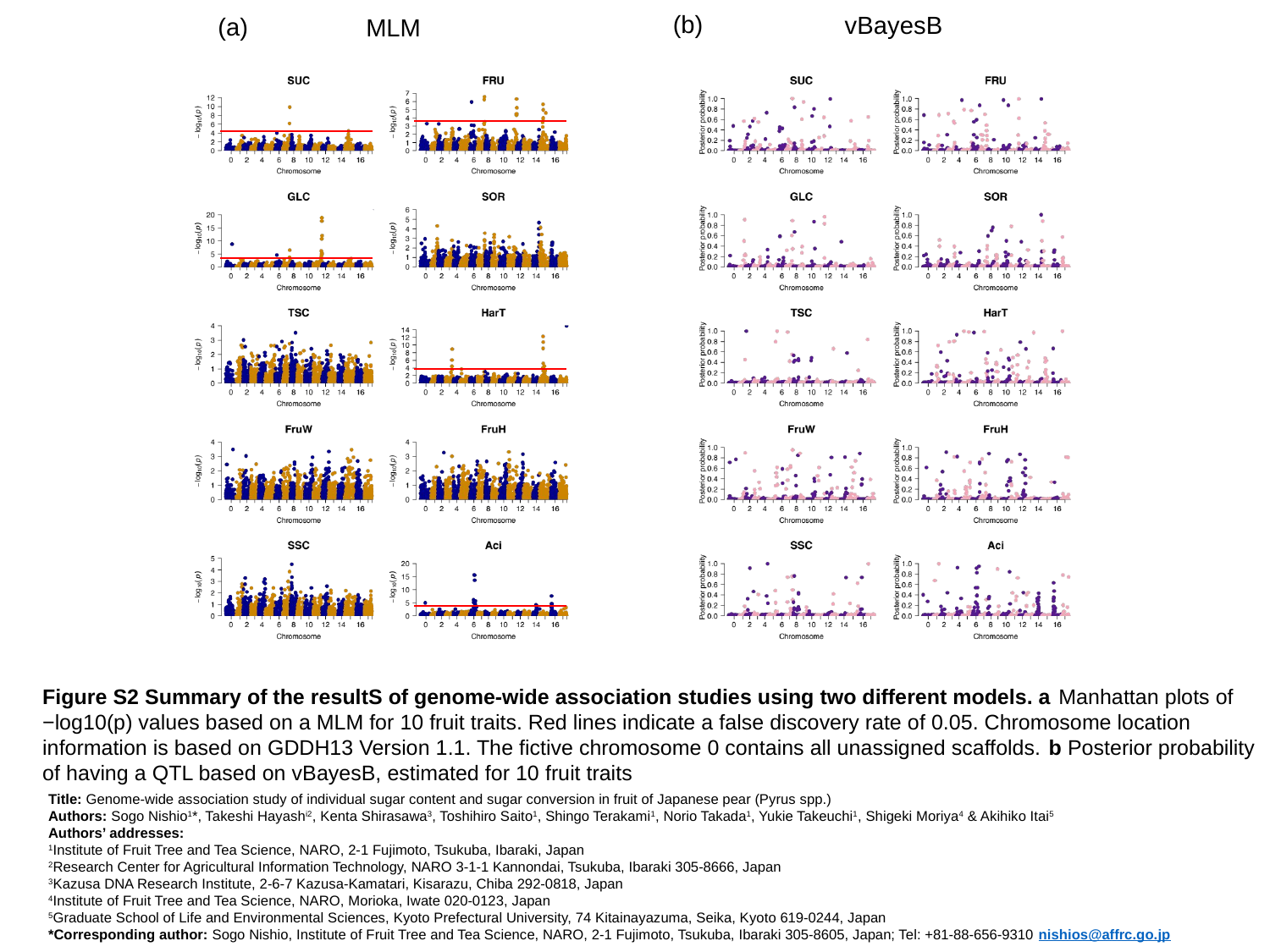

(b)
vBayesB
(a)
MLM
Figure S2 Summary of the resultS of genome-wide association studies using two different models. a Manhattan plots of −log10(p) values based on a MLM for 10 fruit traits. Red lines indicate a false discovery rate of 0.05. Chromosome location information is based on GDDH13 Version 1.1. The fictive chromosome 0 contains all unassigned scaffolds. b Posterior probability of having a QTL based on vBayesB, estimated for 10 fruit traits
Title: Genome-wide association study of individual sugar content and sugar conversion in fruit of Japanese pear (Pyrus spp.)
Authors: Sogo Nishio1*, Takeshi Hayashi2, Kenta Shirasawa3, Toshihiro Saito1, Shingo Terakami1, Norio Takada1, Yukie Takeuchi1, Shigeki Moriya4 & Akihiko Itai5
Authors’ addresses:
1Institute of Fruit Tree and Tea Science, NARO, 2-1 Fujimoto, Tsukuba, Ibaraki, Japan
2Research Center for Agricultural Information Technology, NARO 3-1-1 Kannondai, Tsukuba, Ibaraki 305-8666, Japan
3Kazusa DNA Research Institute, 2-6-7 Kazusa-Kamatari, Kisarazu, Chiba 292-0818, Japan
4Institute of Fruit Tree and Tea Science, NARO, Morioka, Iwate 020-0123, Japan
5Graduate School of Life and Environmental Sciences, Kyoto Prefectural University, 74 Kitainayazuma, Seika, Kyoto 619-0244, Japan
*Corresponding author: Sogo Nishio, Institute of Fruit Tree and Tea Science, NARO, 2-1 Fujimoto, Tsukuba, Ibaraki 305-8605, Japan; Tel: +81-88-656-9310 nishios@affrc.go.jp

## Slide 3
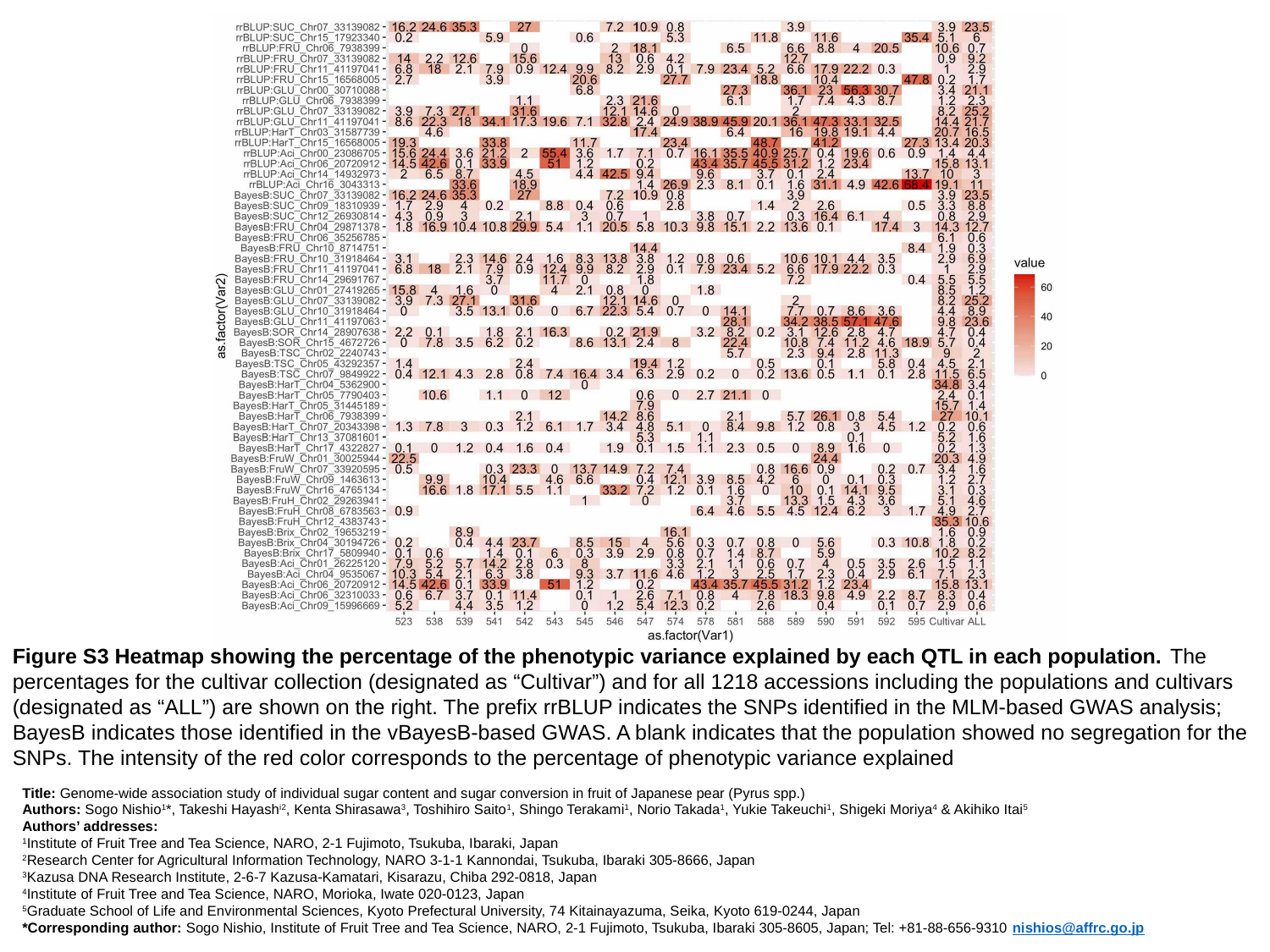

Figure S3 Heatmap showing the percentage of the phenotypic variance explained by each QTL in each population. The percentages for the cultivar collection (designated as “Cultivar”) and for all 1218 accessions including the populations and cultivars (designated as “ALL”) are shown on the right. The prefix rrBLUP indicates the SNPs identified in the MLM-based GWAS analysis; BayesB indicates those identified in the vBayesB-based GWAS. A blank indicates that the population showed no segregation for the SNPs. The intensity of the red color corresponds to the percentage of phenotypic variance explained
Title: Genome-wide association study of individual sugar content and sugar conversion in fruit of Japanese pear (Pyrus spp.)
Authors: Sogo Nishio1*, Takeshi Hayashi2, Kenta Shirasawa3, Toshihiro Saito1, Shingo Terakami1, Norio Takada1, Yukie Takeuchi1, Shigeki Moriya4 & Akihiko Itai5
Authors’ addresses:
1Institute of Fruit Tree and Tea Science, NARO, 2-1 Fujimoto, Tsukuba, Ibaraki, Japan
2Research Center for Agricultural Information Technology, NARO 3-1-1 Kannondai, Tsukuba, Ibaraki 305-8666, Japan
3Kazusa DNA Research Institute, 2-6-7 Kazusa-Kamatari, Kisarazu, Chiba 292-0818, Japan
4Institute of Fruit Tree and Tea Science, NARO, Morioka, Iwate 020-0123, Japan
5Graduate School of Life and Environmental Sciences, Kyoto Prefectural University, 74 Kitainayazuma, Seika, Kyoto 619-0244, Japan
*Corresponding author: Sogo Nishio, Institute of Fruit Tree and Tea Science, NARO, 2-1 Fujimoto, Tsukuba, Ibaraki 305-8605, Japan; Tel: +81-88-656-9310 nishios@affrc.go.jp
